# The functional change of the P2X7R containing the Ala^348^ to Thr polymorphism is associated with the pathogenesis of gout

**DOI:** 10.1038/s41598-023-32365-2

**Published:** 2023-04-05

**Authors:** Man-Yun Li, Xuan Fang, Yan Ma, Xian-Yang Pan, Xiao-Juan Dai, Xiao-Mei Li, Xiao-Ling Li, Yi-Ping Wang, Jin-Hui Tao, Xiang-Pei Li

**Affiliations:** 1 Anhui Provincial Children’s Hospital, Children’s Hospital of Fudan University Anhui Hospital, Hefei, Anhui Province 230051 People’s Republic of China; 2 The First Affiliated Hospital of USTC, Division of Life Sciences and Medicine, University of Science and Technology of China, Hefei, Anhui Province 230001 People’s Republic of China; 3 Centre for Transplantation and Renal Research, Westmead Institute for Medical Research, The University of Sydney, Sydney, NSW Australia

**Keywords:** Cell biology, Genetics, Immunology, Rheumatology

## Abstract

Our previous study has shown that ATP action on P2X7R could be the second signal to induce the onset of gouty arthritis. However, the functional changes of P2X7R single nucleotide polymorphisms (SNPs) on the effects of ATP-P2X7R-IL-1β signaling pathway and uric acid remained unknown. We aimed to investigate the association between the functional change of P2X7R containing the Ala^348^ to Thr polymorphisms (rs1718119) and the pathogenesis of gout. First, 270 gout patients and 70 hyperuricemic patients (without gout attack history in recent 5 years) were recruited for genotyping. In addition, the changes of ATP-induced pore formation were assessed in HEK-293T cells overexpressing different mutants in *P2RX7*, and the effects on P2X7R-NLRP3-IL-1β pathway activation were explored in *P2RX7* overexpression THP-1 cells. The risk allele for gout was A at rs1718119, and the AA and AG genotypes exhibited a higher risk of gout. Furthermore, Ala^348^ to Thr mutants increased P2X7-dependent ethidium^+^ bromide uptake, upregulated IL-1β and NLRP3 levels as compared to the wild-type. We suggest that genetic polymorphisms of P2X7R containing the Ala^348^ to Thr are associated with the increased risk of gout, showing an enhanced gain-of-function effect on the development of this disease.

## Introduction

Gout is an inflammatory arthritis caused by hyperuricemia. Supersaturated blood uric acid forms monosodium urate (MSU) crystals that are deposited in the tissues surrounding the joints, causing joint swelling and pain^1^. Emerging evidence have highlighted that MSU crystals could induce the onset and development of gout through affecting the activation and signal transduction of pyrin domain containing 3 (NLRP3), toll-like receptors (TLRs) and the oligomerization domain (NOD)-like receptor to produce interleukin-1beta (IL-1β). The NLR family contains two inflammatory bodies, NLRP3 and absent in melanoma 2 (AIM2). NLRP3 enables to recruit caspase-1 in an ASC-dependent manner^2^. After recognition of specific agonists, NLRP3 and AIM2 form inflammasomes. Therefore, ASC is an inflammasome adapter protein that is required for the formation of the AIM2 and NLRP3 inflammasomes^3^. In addition, caspase-1 also plays a critical role as a mediator that activates the subsequent maturation of the pro-inflammatory cytokine IL-1β^4^.

In the process of IL-1β secretion induced by the NLRP3 inflammasome stimulated by MSU, IL-1β cannot be released into extracellular through the classical secretion pathway because of the lack of a signal sequence needed for exocrine secretion through the endoplasmic reticulum/Golgi pathway^5^. Recent studies have found that Gasdermin D (GSDMD), as a novel discovered perforating protein, could form pores in the cell membrane, and plays an important role in mediating the secretion and release of IL-1β, as well as pyroptosis. The activation of NLRP3 inflammasome enables to induce the caspase-1 to cleave pro-IL-1β and pro-GSDMD into IL-1β and GSDMD, the aggregates of GSDMD play a key role in the promotion of mature IL-1β secretion^6^. When pyroptosis occurs, ATP is released from the intracellular to extracellular space and is bound to participate in the inflammatory process of gout. Therefore, we previously proposed that the P2X7R receptor for ATP is a key factor regulating the gout pathogenesis^1^.

P2X7R is an ATP-gated ion channel that supports the influx of Na^+^ and Ca^2+^ and the efflux of K^+^ from the cytoplasm^7^. P2X7R stimulation with high (0.5–1 mM and above) concentrations of ATP results in the opening of a nonselective pore permeable to hydrophilic solutes up to 900 Da, such as ethidium^+^ bromide, Yo-Pro, or Lucifer yellow^8–10^. P2X7R, as one of the most potent activators, could be activated by extracellular ATP to induce NLRP3 inflammasome assembly^11^.

A number of single nucleotide polymorphisms (SNPs) have been identified in the region of human *P2X7R* and *P2RX7* gene, such as rs2230911, rs3751143, rs208294, et al.^12–15^, which can affect K^+^ efflux and change the functional status of P2X7R by affecting pore formation ability^13^. Since most patients with hyperuricemia do not develop gout attacks for life, the strength of P2X7R function determines whether patients with hyperuricemia will develop gout. Lee et al.^16^ found that the rs3751142 in *P2RX7* gene is associated with susceptibility to gout. Our previous research also found that rs1653624, rs7958316 and rs17525809 in *P2RX7* gene are associated with susceptibility to gouty arthritis^13^.

The function of P2X7R was determined by multiple SNPs. Gong et al.^12^ revealed that the SNPs in *P2RX7* were related to IL-1β secretion, and considered that these SNPs, such as Ala^348^ to Thr (rs1718119), His^155^ to Tyr (rs208294), Glu^496^ to Ala (rs3751143), Arg^307^ to Gln (rs28360457) and Thr^357^ to Ser (rs2230911), were related to the pathogenesis of gout. For instance, the Ala^348^ to Thr polymorphism (rs1718119) at position 1068 of the *P2RX7* gene from single base substitutions has been identified in exon 11 located in transmembrane domain 2. The Ala^348^ to Thr polymorphism exhibits increased P2X7R functional responses. Stimulation of human monocytes from individuals carrying gain-of-function of the *P2RX7* gene induces increased secretion of the proinflammatory cytokine IL-1β when compared to wild-type^13^. However, Ying^14^ and our previous study^13^ did not find an association between rs1718119 in *P2RX7* and the development of gout in Chinese individuals.

Since the abundant SNPs carried by the *P2RX7* gene affect its functional development, studies in different study populations may draw different conclusions. Therefore, the Ala^348^ to Thr polymorphism (rs1718119) was reanalyzed in our study, and we further transfected THP-1 cells with lentivirus. In order to further compare the functional differences between wild-type and the Ala^348^ to Thr polymorphism in vivo with high uric acid. In our study, the allele and genotype frequencies of rs1718119 were found to be associated with gout susceptibility. The A allele and GG/(AA + AG) genotypes in rs1718119 were at a higher risk of gout, and the Ala^348^ to Thr polymorphism has the major effect increased receptor function via ATP induced with high uric acid.

## Materials and methods

### Subjects and collection of clinical information

This was designed as a case‒control study, and a total of 270 gout patients were recruited for the current study. All gout patients were diagnosed according to the American College of Rheumatology classification criteria^17^. In the present study, we only selected male cases because male individuals were more susceptible than females to developing gout^18^.

A total of 70 people with elevated blood uric acid levels, defined as patients with hyperuricemia (disease duration of more than 5 years, serum uric acid level > 480 μmol/L (8 mg/dL) and no history of gout attacks), were recruited from medical examination centers or related institutions. This is based on studies that have shown that urate levels are associated with gout and gout progression over a 5-year period^19^. Two groups of genomic DNA samples were extracted from peripheral venous blood from 270 gout patients and 70 hyperuricemia patients using a Qiagen DNA Kit (Qiagen, Germany) following the standard DNA isolation instructions.

### Selection of SNPs loci of P2X7R

In the process of searching for suitable point mutation sequences from the human P2X7R cDNA sequence (GenBank accession number: NM_002562.5), Ala^348^ to Thr (rs1718119), which was believed to impact the secretion of IL-1β and thus play a vital role in the pathogenesis of gout^12^, was selected and examined in our present study. The SNPs were genotyped using an EP1™ high-throughput gene analysis system (Fluidigm, USA). The linkage disequilibrium (LD) between selected SNPs were calculated based on HapMap genotype data using PLINK software version 1.07^20^. In addition, a THP-1-cell model transfected with the Ala^348^ to Thr polymorphism was constructed.

### Cell culture

HEK-293T cells (Shanghai Institute of Cell Biology, Chinese Academy of Sciences, Shanghai, China) were cultured in Dulbecco’s modified Eagle’s medium containing high glucose (4.5 g/L) and GlutMAXTM I (3.97 mM) (DMEM, Gibco) supplemented with 5% fetal bovine serum (FBS, Hyclone). THP-1 cells were a gift from the Department of Immunology, Shanghai Institute of Cell Biology, Chinese Academy of Sciences. The cells were grown in RPMI-1640 medium (Gibco) supplemented with 10% heat-inactivated FBS (Gibco) and cultured at 37 °C and 5% CO_2_ in an incubator. THP-1 and HEK-293T cells were placed in 6-well plates at a density of 1.0–1.5 × 106/mL and passaged again every 2–3 days when the cell population was close to 80–90% confluence.

### Lentivirus production and transduction

Lentivirus was generated for overexpression of P2X7R in HEK-293T cells and THP-1 cells. Lentivirus vectors were generated by inserting the P2X7R sequences encoding human P2X7R into the multi-cloning site of the lentivirus backbone plasmid pHBLV-CMV-MCS-3FLAG-EF1-ZsGreen-T2A-PURO. The constructs were co-transfected with packaging vectors into HEK-293T cells for packaging followed by purification (Hanbio Biotechnology Co., LTD, Shanghai, CHINA). Then, HEK-293T cells and THP-1 cells were infected with concentrated virus solution. Cells were used for further assays 3 days after transfection. HEK-293T cells were transduced at a MOI of 10, and THP-1 cells were transduced at a MOI of 100. Cells were transduced with a lentiviral overexpression vector containing P2X7R lentiviral particles in the presence of 6 μg/mL polybrene. After 24 h, the culture medium was removed, and fresh medium was added. After transfection, stably transfected HEK-293T or THP-1 cells were obtained for 1 day or 3 days, respectively.

### Ethidium influx measurement

The effect of different mutations of P2X7R on pore formation after ATP induction was assessed by flow cytometry (BD Biosciences, San Jose, USA) to detect the uptake of ethylenediamine + bromide by HEK-293T cells. Cells (1 × 10^5^) prelabeled with GFP-conjugated LVs were washed once and resuspended in 1.0 mL of HEPES-buffered KCl medium (150 mM KCl, 5 mM d-glucose, 0.1% bovine serum albumin, 10 mM HEPES, pH 7.5) at 37 °C. All samples were stirred and temperature-controlled at 37 °C using a Time Zero module. Ethidium^**+**^ (25 µM) was added, followed 40 s later by the addition of 1.0 mM ATP. Cells were analyzed at 1000 events/s by flow cytometry. The linear mean channel of fluorescence intensity (0–255 channel) for each gated subpopulation over successive 5-s intervals was analyzed by the Flwojo software and plotted against time^21^. All experiments in this research were performed in triplicate independently.

### Functional experiment

THP-1 cells were plated at a density of 2.5 × 10^4^ in 24-well plates and then stimulated for 3 h with 100 ng/mL PMA (Sigma) the day before stimulation^22^. This treatment enhances the phagocytic properties of the cells and prompts constitutive production of pro-IL-1β^23,24^. The purchased MSU (Sigma) crystals were dissolved with sodium hydroxide to prepare a MSU emulsion at a concentration of 100 μg/mL to stimulate THP-1 cells. Finally, THP-1 cells were randomly divided into three groups: MSU (labeled as group M), MSU + ATP (labeled as group MA), and unstimulated control (labeled as group C). Groups M and MA were stimulated by adding MSU for 24 h, followed by ATP (Sigma) stimulation in the MA group for 60 min.

### Enzyme-linked immunosorbent assay

Samples were obtained from the supernatant of THP-1 cells. The presence of IL-1β in the supernatant was measured with commercial enzyme-linked immunosorbent assay (ELISA) kits (R&D Systems, Minnesota, USA) according to the manufacturer’s instructions. The absorbance was read at 450 nm using a microplate reader. All experiments in this research were performed in triplicate independently.

### Quantitative real-time polymerase chain reaction (qRT‒PCR)

Total RNA was isolated in using TRIzol reagent (Sigma), and the concentration of RNA was determined using a NanoDrop 2000 (Thermo Fisher Scientific, Wilmington, DE, USA). RNA was reverse-transcribed with a cDNA Reverse Transcription Kit (QIAGEN, Duesseldorf, Germany), after which quantitative real-time PCR amplification was performed with a SYBR® Green PCR Kit (Qiagen) according to the manufacturer’s instructions. Primers for human IL-1β, NLRP3, ASC, and caspase-1 were synthesized by Sangon Biotech (Shanghai, China). The sequences of the primers are shown in Table 1. All cDNA samples were amplified in the ABI 7500 Fast Real-Time PCR System (Applied Biosystems, California, USA). Data were analyzed using the ^ΔΔ^Ct comparative quantification method following normalization to β-actin. All experiments in this research were performed in triplicate independently.

**Table 1:** PCR primers.

P2X7	Forward primer 5′-TCAGACCGGAAGGTG-3′
	Reverse primer 5′-TCAGACCGGAAGGTG-3′
IL-1β	Forward primer 5′-TGGCTTATTACAGTGGCAATGAGG-3′
	Reverse primer 5′-AGTGGTGGTCGGAGATTCGTAG-3′
NLRP3	Forward primer 5′-GATCTTCGCTGCGATCAACAG-3′
	Reverse primer 5′-CGTGCATTATCTGAACCCCAC-3′
ASC	Forward primer 5′-CGTTGAGTGGCTGCTGGATG-3′
	Reverse primer 5′-CAGGCTGGTGTGAAACTGAAGAG-3′
caspase-1	Forward primer 5′-ACACCGCCCAGAGCACAAG-3′
	Reverse primer 5′-TTTCTTCCCACAAATGCCTTCCC-3′
β-actin	Forward primer 5′-CCTTCCTGGGCATGGAGTCCTG-3′
	Reverse primer 5′-GGAGCAATGATCTTGATCTTC-3′

### Statistical analysis

Data were analyzed using SPSS 23.0 software (SPSS, Chicago, IL, USA). and the graphics were drawn by GraphPad Prism 6. The *P* value < 0.05 was considered statistically significant. To determine statistical significance among multiple comparisons, a one-way ANOVA followed by a post hoc analysis was used. Differences between two individual experimental groups were compared by a two-tailed *t* test. In addition, we compared the SNPs genotype and allele frequencies between the gout and hyperuricemia groups for each SNPs. Odds ratios (ORs) and 95% confidence intervals (CIs) were calculated using nonconditional logistic regression analyses. Quantitative data are presented as the mean ± SD. Hardy–Weinberg equilibrium (HWE) in gout patients and normal controls was determined using SHEsis software^24^.

### Ethics declarations

This study conformed to the principles expressed in the Declaration of Helsinki. All subjects gave their written informed consent as approved by the respective ethical committees to participate in the study, which was approved by the Medical Ethics Committee of the First Affiliated Hospital of USTC, Division of Life Sciences and Medicine, University of Science and Technology of China (2021 KY London Examination No. 155).

### Informed consent

Current study was conducted by the First Affiliated Hospital of the University of Science and Technology of China and the Department of Life Sciences and Medicine of the University of Science and Technology of China. Informed consent was obtained from all enrollees (a total of 270 gout patients and 70 hyperuricemic patients). And informed consent from these participants with their information or images also have been obtained to publish their information or images.

## Results

### Demographic characteristics

A number of 270 male gout patients and 70 male hyperuricemia subjects were enrolled. The mean ages of gout and hyperuricemia subjects were 52.7 ± 16.2 and 49.4 ± 13.4 years old, respectively. The mean body mass index (BMI) for gout patients and hyperuricemia subjects were 26.7 ± 0.6 and 25.9 ± 0.4, respectively. There were no significant differences in age and BMI between these two groups (*P* > 0.05) (Table 2).

**Table 2:** Age, height, weight, body mass index, and sex ratios at presentation between the two groups.

rs1718119	GOUT	Hyperuricemia	*P*-value
Age, years, Mean ± SD	52.7 ± 16.2	49.4 ± 13.4	0.29
Height, cm, Mean ± SD	171.3 ± 0.7	170.7 ± 0.5	0.50
Weight, kg, Mean ± SD	78.5 ± 2.0	78.8 ± 1.26	0.24
body mass index, Mean ± SD	26.7 ± 0.6	25.9 ± 0.4	0.29
sex ratios	/	/	/

### Hardy–Weinberg equilibrium test

H-W balance testing for all genotyping results revealed that the rs1718119 genotype frequency was consistent with the HWE equilibrium in Table 3 (*P* > 0.05).

**Table 3:** Hardy–Weinberg equilibrium test of the genotype frequency distribution in the two groups.

SNPs	Group	Genotype			HWE	
		AA	AG	GG	χ^2^	*P*-value
rs1718119	GOUT	16	94	160	0.20	0.658
	Hyperuricemia	2	10	58	2.98	0.084

### Differences in the distribution of SNPs genotypes in P2X7R between gout and hyperuricemia patients

After excluding multiple factors, such as sex, age, height, weight, and body mass index, differences in the prevalence of rs1718119 were observed between gout and hyperuricemia patients. The GG, GA, and AA genotype frequencies of rs1718119 were 82.9%, 14.3%, and 2.9%, respectively, in the hyperuricemia group and 59.3%, 34.8%, and 5.9%, respectively, in the gout group. There was a statistically significant difference between the two groups in rs1718119 genotype distribution (χ^2^ = 13.48, *P* = 0.001). In the hyperuricemia group, the frequencies of the A and G alleles were 10.0% and 90.0%, respectively, which were 23.3% and 76.7%, respectively, in the gout group. The gout-sensitivity allele at rs1718119 was A (OR = 2.74, 95% CI: 1.50–5.33). The AA and AG genotypes exhibited a higher risk of gout (AG vs. GG, OR = 3.41 (95% CI: 1.62–7.82); (AA + AG) vs. GG, OR = 3.33 (95% CI: 1.66–7.10)). Although the comparison between gout and hyperuricemia patients carrying the recessive genetic model (AG + GG) showed a slightly increased odds ratio [OR = 2.14, 95% CI (0.48–19.62)], the difference was not significant, as shown in Table 4 (*P* = 0.307).

**Table 4:** Allele and genotype frequencies and genetic model of SNPs rs1718119 in the two groups.

Genetic model	GOUTs	Hyperuricemias	χ^2^	*P*-value	OR	95% CI
A vs. G	126/414	14/126	12.09	< 0.001	2.74	1.50–5.33
AA vs. GG	16/160	2/58	2.11	0.147	2.90	0.65–26.68
AG vs. GG	94/160	10/58	12.20	< 0.001	3.41	1.62–7.82
(AA + AG) vs. GG	110/160	12/58	13.45	< 0.001	3.33	1.66–7.10
AA vs. (AG + GG)	16/254	2/68	1.04	0.307	2.14	0.48–19.62

### Association between the number of susceptible genotypes and the risk of gout

According to several previous studies, the P2X7R SNPs (rs1718119) was the dominant gene. Homozygous and heterozygous hyperuricemia patients with this allele exhibit a higher risk of gout than patients without this allele. Patients with hyperuricemia may have a relatively low risk of gout if they carry a single SNP-susceptible genotype, but if they carry more than one susceptible SNPs, the risk of gout may be increased. Based on this hypothesis, we further analyzed the risk of gout in patients with hyperuricemia who carry the susceptible SNPs genotypes. The results demonstrated that more susceptible genotypes increased gout risk, and the OR reached 4.91 (95% CI: 2.34–11.23) in patients with susceptible genotypes, as shown in Table 5.

**Table 5:** Association between the number of susceptible genotypes and the risk of gout.

Contains all gout susceptibility genotypes?	Gout (n)	Hyperuricemia (n)	*P*-value	OR	95% CI
Yes	108	10	< 0.001	4.91	2.34–11.23
No	132	60			

### Dominant positive effect of Ala^348^ to Thr on P2X7 pore formation function in HEK-293T cells

**Figure 1:**
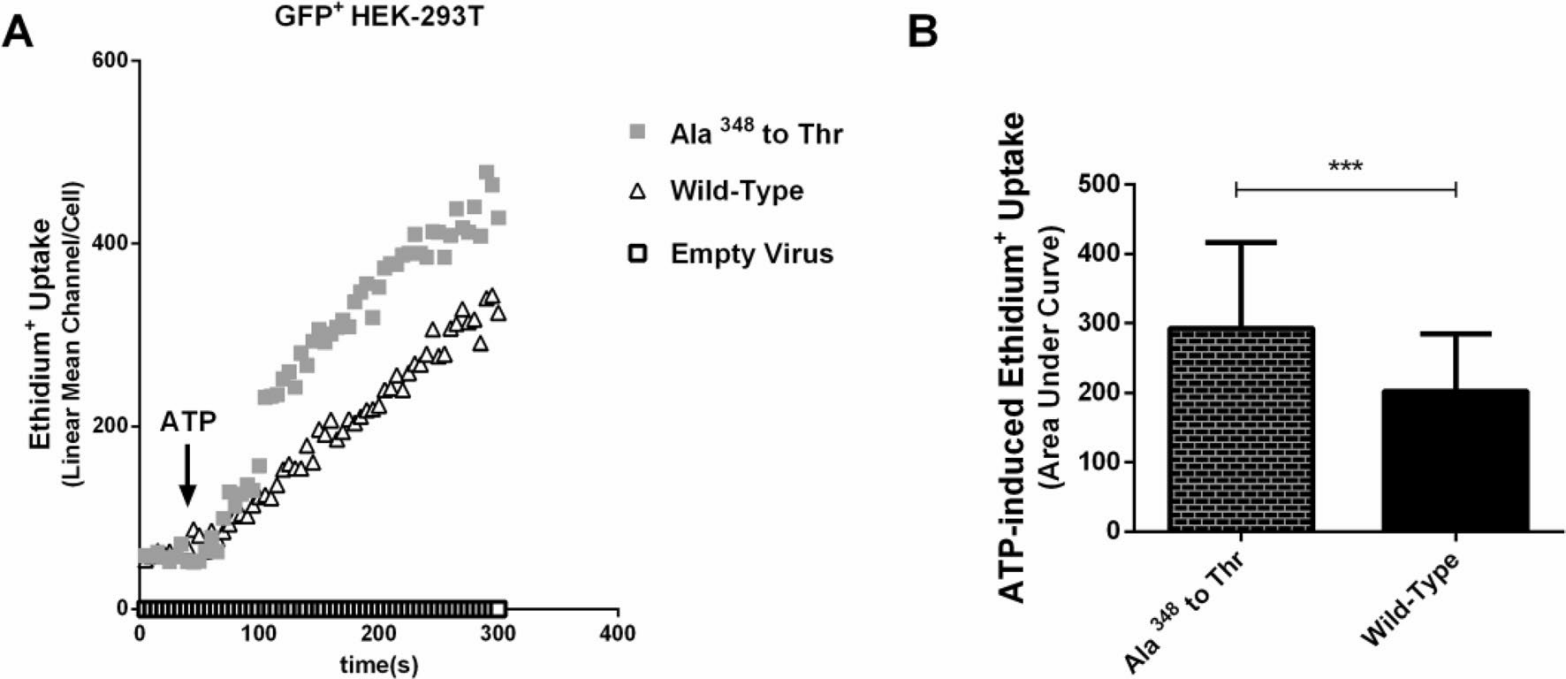
The functional effect of Ala^348^ to Thr in transfected HEK-293T cells. (**A**) P2X7-dependent ethidium^+^ uptake induced by 1 mM ATP (applied at arrow) in Ala^348^ to Thr compared with Wild-Type. (**B**) Mean ATP-induced ethidium^**+**^ bromide uptake was quantified by calculating the area under the dye uptake curve for cells expressing Ala^348^ to Thr. ***P < 0.001.

HEK-293T cells were transfected with a lentivirus carrying the P2X7R containing the Ala^348^ to Thr polymorphism. Non-transfected cells (empty virus) were used as a blank control, and transfected cells (wild-type) were used as an experimental control. We examined the functional effect of Ala^348^ to Thr on P2X7 pore formation using an ATP-induced ethidium^+^ bromide uptake assay. Cells were stimulated with ATP for 260 s. The wild-type showed brisk uptake of ethidium^+^ bromide dye via P2X7R. The Ala^348^ to Thr mutation showed P2X7-mediated ethidium^+^ bromide uptake, which was greater than the wild-type value in Fig. 1A.

The Ala^348^ to Thr polymorphism has a gain-of-function effect on the uptake function of ATP-induced ethidium^+^ bromide. It increased P2X7-dependent ethidium^**+**^ bromide uptake, as shown in Fig. 1B (145% of the wild-type P2X7 response, *P* < 0.001).

### Establishment of a THP-1 cell line stably expressing the P2X7R containing wild-type and Ala^348^ to Thr

**Figure 2:**
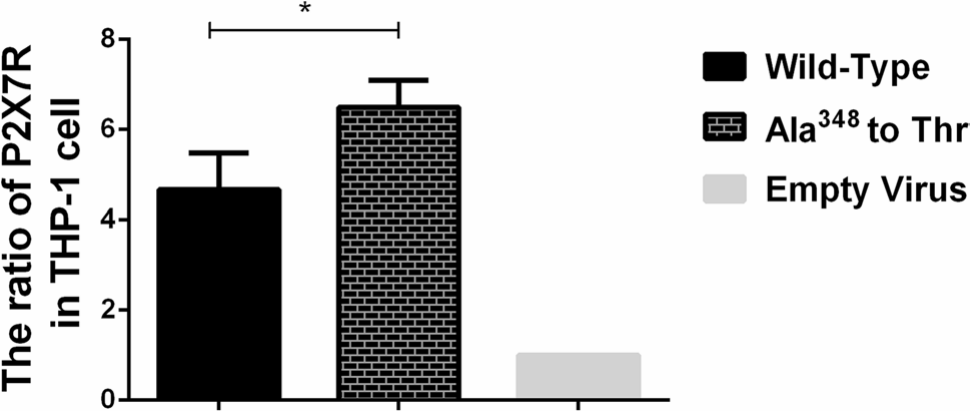
mRNA expression levels of P2X7R in THP-1 cells carrying Ala^348^ to Thr, Wild-Type and Empty Virus. *P < 0.05.

As shown in Fig. 2, lentivirus carrying the Ala^348^ to Thr mutation, wild-type or empty virus was stably transferred into THP-1 cells. The mRNA expression of P2X7R in cells with the Ala^348^ to Thr mutation was higher than that in the wild-type cells (*P* = 0.035).

### The Ala^348^ to Thr mutation enhanced the expression of IL-1β and NLRP3

**Figure 3:**
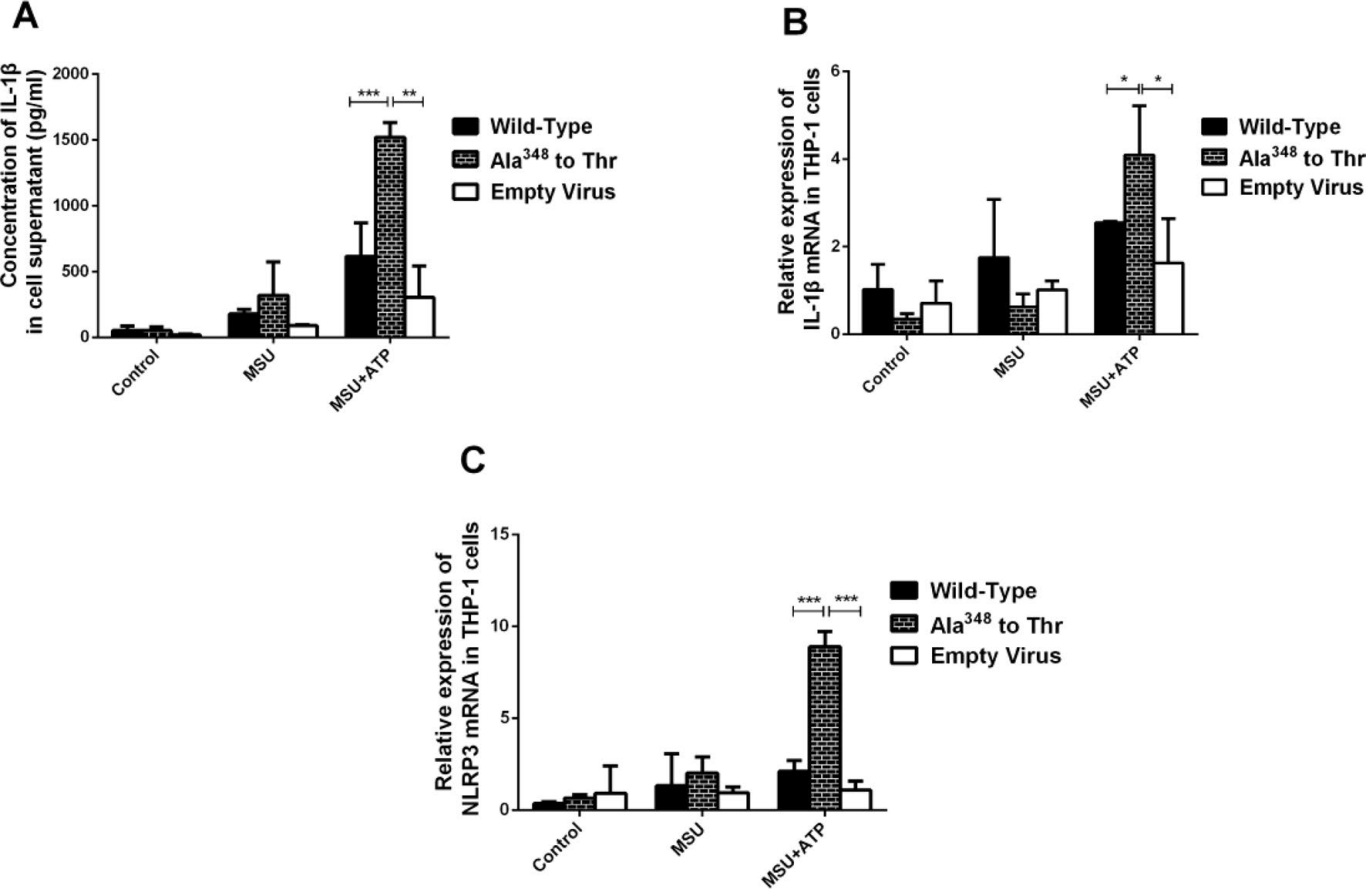
IL-1β and NLRP3 levels with the Ala^348^ to Thr mutation. (**A**) IL-1β levels in Ala^348^ to Thr, Wild -Type and Empty Virus in the supernatant. (**B**) IL-1β mRNA expression in the Ala^348^ to Thr, Wild-Type and Empty Virus groups. (C) NLRP3 mRNA expression after treatment with Ala^348^ to Thr, Wild-Type or Empty Virus. *P < 0.05, **P < 0.01, ***P < 0.001.

Initially, the stimulation of THP-1 cells with Phorbol-12-myristate-13-acetate (PMA), MSU and ATP induced a strong increase in IL-1β levels. THP-1 cells were transfected with a lentivirus carrying Ala^348^ to Thr mutation, wild-type, or empty virus. Cells were then stimulated with or without MSU or MSU + ATP. The supernatant and cells were collected to detect the levels of IL-1β and NLRP3. In the MA group, the Ala^348^ to Thr mutation significantly up-regulated the levels of IL-1β compared with the wild-type and empty virus (*P* = 0.0007; *P* = 0.013, respectively). The wild-type had higher levels of IL-1β than the empty virus, but with no statistical significance (*P* > 0.05) in Fig. 3A. Moreover, the mRNA expression of IL-1β in cells with the Ala^348^ to Thr mutation was higher than that in cells infected with the wild-type and empty virus (*P* = 0.0334 and *P* = 0.0307, respectively) (Fig. 3B). The mRNA expression of NLRP3 in cells with the Ala^348^ to Thr mutation was also higher than that in cells infected with the wild-type and empty virus (*P* = 0.0003 and *P* = 0.0001, respectively) (Fig. 3C). However, the levels of IL-1β and NLRP3 among the Ala^348^ to Thr, wild-type and empty virus groups in groups C and M were not significantly different, as shown in Fig. 3 (*P* > 0.05).

### ASC and Caspase-1 were not affected by the Ala^348^ to Thr mutation

**Figure 4:**
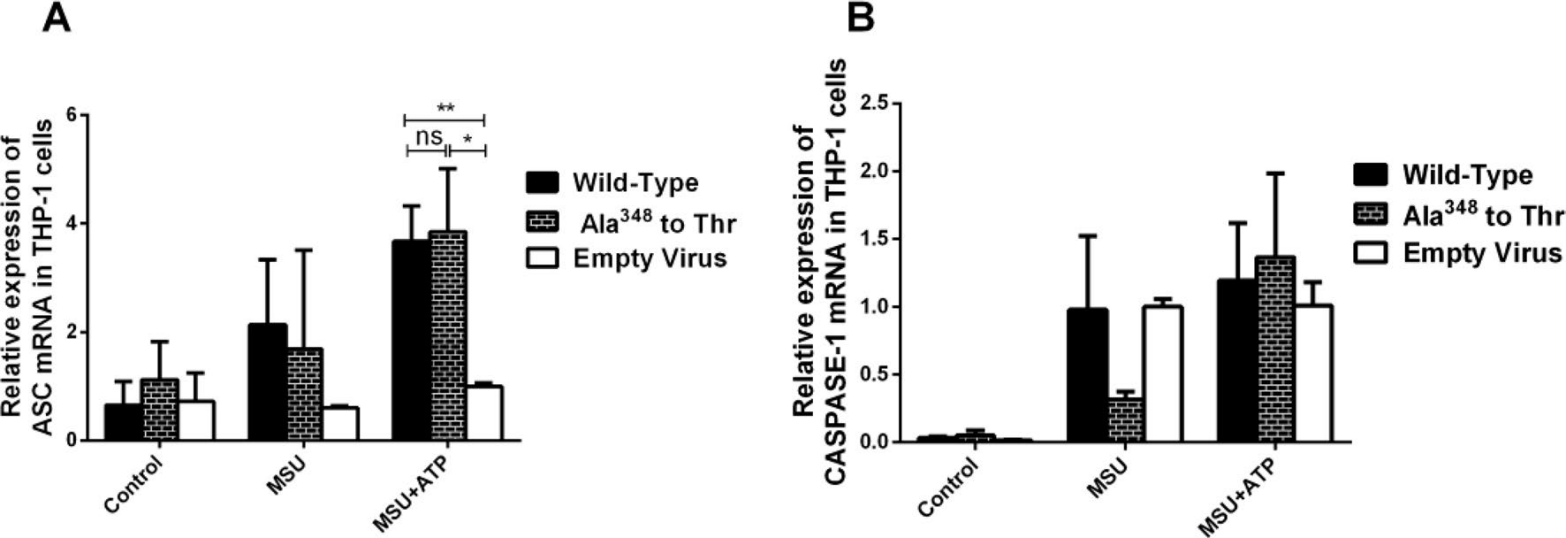
The ASC and Caspase-1 levels in the Ala^348^ to Thr mutation. (**A**) ASC mRNA expression in Ala^348^ to Thr, Wild-Type and Empty Virus. (**B**) Caspase-1 mRNA expression in Ala^348^ to Thr, Wild-Type and Empty Virus groups. *P < 0.05, **P < 0.01.

As shown in Fig. 4, unlike IL-1β and NLRP3 gene expression, no statistical significance was found in the mRNA expression of ASC and caspase-1 among Ala^348^ to Thr, wild-type and empty virus in all three groups (*P* > 0.05).

## Discussion

P2X7R is highly expressed in almost all tissues and organs, especially in immune cells of monocyte-macrophage origin^25^. The expression and function of P2X7R are affected by the concentration of extracellular ATP. A study showed that dilatation of the P2X7R channel forms a “pore” stimulated by extracellular ATP, which is recognized as a unique feature of P2X7R^26^. Prolonged or repeated exposure to the P2X7R ligand opens a non-selective cation channel, followed by the formation of a cytolytic pore permeable to allow NLRP3 agonists to enter the cytoplasm to activate the NLRP3 inflammasome^27,28^. Because there are many SNPs in P2X7R, different genotypic changes in some of the non-synonymous coding sites can change the types of amino acids. The amino acid sequence of P2X7R is affected, which in turn affects the function of P2X7R. Two studies^29,30^ showed that the rs1718119 polymorphism of P2X7R was associated with Systemic lupus erythematosus (SLE) and Rheumatoid arthritis (RA). It is suggested that the functional changes of P2X7R in different genotypes of rs1718119 (Ala^348^ to Thr) can affect the occurrence of related diseases.

In contrast to the results of previous studies on the relationship between the P2X7R rs1718119 gene polymorphism and susceptibility to gout^13,14^, we found that the rs1718119 gene polymorphism is associated with gout susceptibility. After excluding multiple factors, such as sex, age, height, weight, and body mass index, the reason for this difference may be that the function of P2X7R is affected by multiple SNPs. In the individual, the combined action of multiple SNPs determines the susceptibility to gout. In hyperuricemia patients with rs1718119, if they also carry the insusceptible genotype of other SNPs, gout may not occur. Therefore, the results may show that it is not associated with gout. For example, we previously found that the risk of gout increased significantly when multiple P2X7R SNPs were carried simultaneously^13^. In addition, Ying^14^ found that there was linkage disequilibrium in rs1718119. In patients with gout, if multiple SNPs are not susceptible to genotype linkage to rs1718119, it can also obscure the association between the rs1718119 gene polymorphism and the pathogenesis of gout.

Stokes^15^ has shown a significant association between massive K^+^ loss from the cell and P2X7R pore dilatation, largely due to the presence of the Ala^348^ to Thr mutation. Because the function of ATP-stimulated P2X7R detected by the amount of ethidium^+^ bromide and the occurrence of gout must have the participation of MSU, we further investigated the effect of different rs1718119 genotypes on P2X7R function at the onset of gout and conducted related experiments with high uric acid. As shown in our results, our findings are consistent with previous studies. In our research, transfection of the Ala^348^ to Thr mutant in HEK-293T cells increased ATP-induced P2X7-dependent ethidium^+^ bromide uptake to 145% of that of the wild-type. After antagonizing ATP, it showed that P2X7R was activated in Ala^348^ to Thr mutant and wild-type, and we observed the strength of P2X7R function by detecting the amount of ethidium^+^ bromide uptake by HEK-293T cells. We further found that THP-1 monocytic cells with P2X7R carrying Ala^348^ to Thr increased ATP-induced secretion of the proinflammatory cytokine IL-1β, this is consistent with previous study, where Marinho et al.^31^ revealed that MSU crystals induced sterile IL-1β secretion via P2X receptor activation. The expression level of the NLRP3 gene in THP-1 cells expressing the Ala^348^ to Thr mutant via ATP stimulation was significantly increased than that of the wild-type and empty virus groups, it suggested that the Ala^348^ to Thr polymorphisms showed a dominant effect in conferring an enhanced P2X7R function, and could upregulate inflammation via ATP-stimulated P2X7R with high uric acid levels. The above in vitro findings were consistent with our previous part regarding the SNPs genotypes in *P2RX7* the Ala^348^ to Thr polymorphisms (rs1718119).

NLRP3 inflammasomes can be activated by exogenous (pathogen-associated molecular patterns) PAMPs or endogenous (damage-associated molecular patterns) DAMPs. In addition to MSU, the NLRP3 inflammasome can be activated by many metabolic substances that act as DAMPs in the body, such as ATP, cholesterol, glucose, etc.^32^. Thereinto, extracellular ATP plays an undisputed role^33^. P2X7R is activated by extracellular ATP to activate K^+^ efflux. NLRP3 inflammasome assembly and caspase-1 recruitment occur spontaneously at K^+^ concentrations below 90 mM but are prevented at higher concentrations. Mechanistically, the different signals that stimulate the activation of the NLRP3 inflammasome essentially regulate changes in intracellular potassium, calcium, and other plasma concentrations to activate the NLRP3 inflammasome. Among the many signals that stimulate the activation of the NLRP3 inflammasome, ATP acts through the receptor P2X7R. Due to the direct regulation of P2X7R on the flow of potassium, calcium, and other ions in and out of cells, it is easy to synergize with other pathogenic signals. The ATP-P2X7R signaling pathway can often cooperate with other pathogenic signals to participate in the pathogenesis of diseases. This is also one of the mechanisms by which ATP production can synergize with other pathogenic signals.

Another point to note was that inflammasome complexes required for activation of caspase-1 have been identified^34^. and ASC plays an important role in the activation of NLRP3 and AIM2 inflammasomes as an adaptor protein^25^. Our study showed that the expression of ASC did not change significantly in the wild-type compared to the Ala^348^ to Thr mutation and that the expression of caspase-1 was unchanged significantly between the wild-type and the Ala^348^ to Thr mutation. These results suggest that the ATP-P2X7R signaling pathway activates the NLRP3 inflammasome mainly by upregulating NLRP3 expression. Among them, ASC has a limited role as an adaptor protein. Since the main role of the NLRP3 inflammasome is to cleave inactive pro-caspase-1 into active caspase-1, the expression level of caspase-1 is related to its ability to cleave pro-IL-1β to mature IL-1β, limiting its impact.

In conclusion, we found that the SNPs of P2X7R (rs1718119) altering Ala^348^ to Thr, changed the functions of P2X7R with high uric acid in our study. In addition, the genetic variability in the P2X7R gene with this variant was involved in the process of NLRP3 inflammasome activation. Furthermore, we recently proved that ATP can promote the pathogenesis of gout in mice^35^. All these results further confirmed the key role of P2X7R in regulating the pathogenesis of gout. Due to the up-regulation of CD39 and CD73 expression in the inflammatory environment caused by gout^36^, CD39 and CD73 can gradually degrade ATP into adenosine^37,38^. Since adenosine suppresses inflammation^39^, purine signaling may play a dual role in gout pathogenesis and remission^40^. The functional changes of P2X7R SNPs may be the potential target for the prevention and treatment of gout.

## Data Availability

All data included in this study are available upon request by contact with the corresponding author. No part of this review, including ideas and tables, is copied or published elsewhere.
